# Viral Clearance and Neuroinflammation in Acute TMEV Infection Vary by Host Genetic Background

**DOI:** 10.3390/ijms231810482

**Published:** 2022-09-09

**Authors:** Koedi S. Lawley, Raquel R. Rech, Aracely A. Perez Gomez, Laura Hopkins, Gang Han, Katia Amstalden, C. Jane Welsh, Colin R. Young, Yava Jones-Hall, David W. Threadgill, Candice L. Brinkmeyer-Langford

**Affiliations:** 1 Department of Veterinary Integrative Biosciences, School of Veterinary Medicine and Biomedical Sciences, Texas A&M University, College Station, TX 77843, USA; 2 Department of Veterinary Pathobiology, School of Veterinary Medicine and Biomedical Sciences, Texas A&M University, College Station, TX 77843, USA; 3 Department of Epidemiology and Biostatistics, School of Public Health, Texas A&M University, College Station, TX 77843, USA; 4 Texas A&M Institute for Neuroscience, Texas A&M University, College Station, TX 77843, USA; 5 Department of Molecular and Cellular Medicine, School of Medicine, Texas A&M University, College Station, TX 77843, USA

**Keywords:** collaborative cross, TMEV, acute infection, neurological disease, hippocampal formation, Iba-1, RNA in situ hybridization

## Abstract

A wide range of viruses cause neurological manifestations in their hosts. Infection by neurotropic viruses as well as the resulting immune response can irreversibly disrupt the complex structural and functional architecture of the brain, depending in part on host genetic background. The interaction between host genetic background, neurological response to viral infection, and subsequent clinical manifestations remains poorly understood. In the present study, we used the genetically diverse Collaborative Cross (CC) mouse resource to better understand how differences in genetic background drive clinical signs and neuropathological manifestations of acute Theiler’s murine encephalomyelitis virus (TMEV) infection. For the first time, we characterized variations of TMEV viral tropism and load based on host genetic background, and correlated viral load with microglial/macrophage activation. For five CC strains (CC002, CC023, CC027, CC057, and CC078) infected with TMEV, we compared clinical signs, lesion distribution, microglial/macrophage response, expression, and distribution of TMEV mRNA, and identified genetic loci relevant to the early acute (4 days post-infection [dpi]) and late acute (14 dpi) timepoints. We examined brain pathology to determine possible causes of strain-specific differences in clinical signs, and found that fields CA1 and CA2 of the hippocampal formation were especially targeted by TMEV across all strains. Using Iba-1 immunolabeling, we identified and characterized strain- and timepoint-specific variation in microglial/macrophage reactivity in the hippocampal formation. Because viral clearance can influence disease outcome, we used RNA in situ hybridization to quantify viral load and TMEV mRNA distribution at both timepoints. TMEV mRNA expression was broadly distributed in the hippocampal formation at 4 dpi in all strains but varied between radiating and clustered distribution depending on the CC strain. We found a positive correlation between microglial/macrophage reactivity and TMEV mRNA expression at 4 dpi. At 14 dpi, we observed a dramatic reduction in TMEV mRNA expression, and localization to the medial portion of field CA1 and field CA2. To better understand how host genetic background can influence pathological outcomes, we identified quantitative trait loci associated with frequency of lesions in a particular brain region and with microglial/macrophage reactivity. These QTL were located near several loci of interest: lysosomal trafficking regulator (*Lyst*) and nidogen 1 (*Nid1*), and transmembrane protein 106 B (*Tmem106b).* Together, these results provide a novel understanding about the influences of genetic variation on the acute neuropathological and immunopathological environment and viral load, which collectively lead to variable disease outcomes. Our findings reveal possible avenues for future investigation which may lead to more effective intervention strategies and treatment regimens.

## 1. Introduction

Neurotropic viral infections in humans can cause symptoms which may be mild or acute, but often have long-term consequences for the infected individual. An acute viral infection can feature rapid onset, which may be resolved quickly via innate immune responses of the host and may present with subclinical signs. Alternatively, viral infections may cause severe clinical manifestations resulting in acute or chronic neurological disease conditions. The onset of certain neurological disorders can be connected to viral infections, which may cause symptoms directly or trigger immune responses that contribute to these symptoms. Diseases such as multiple sclerosis [1], amyotrophic lateral sclerosis (ALS) [2], Parkinson’s disease [3], Alzheimer’s disease [4], Guillain Barré syndrome [5], and epilepsy [6] have all been proposed to have a viral trigger [7]. These diseases tend to have presentations that vary between populations, racial groups, and other categories. It is well understood that neurological diseases have a multifactorial etiology, influenced by strong genetic and environmental associations [8]. Mouse models are frequently studied to investigate relationships between host genetic background and susceptibility to virally induced neurological diseases, but these studies have typically involved mice with limited genetic diversity and failed to capture the full spectrum of virally induced neurological disease. 

We use the Collaborative Cross (CC) mouse resource to understand how variation in genetic background leads to diversity in acute behavioral and histological responses to Theiler’s murine encephalomyelitis (TMEV) infection. Briefly, the CC mouse resource consists of mouse strains derived from the multigenerational cross-breeding of eight founder strains (A/J, C57BL/6J, 129S1/SvImJ, NOD/LtJ, NZO/HlLtJ, CAST/EiJ, PWK/PhJ, and WSB/EiJ) [9,10]. The resulting recombinant inbred CC strains carry genetic contributions from each founder. Thus, the CC mouse resource provides a model which more effectively captures the diversity of disease presentation across human populations, compared to classically used inbred mouse strains. The CC mouse resource has also been used as a tractable model for investigations into how host genetics influence pathogen responses [11]. 

TMEV is a naturally occurring murine picornavirus, which can cause different neurological diseases based on the genetic background of the infected mouse strain (i.e., demyelinating disease in SJL/J mice [12,13,14] and epilepsy in C57BL/6J mice [13,14,15]. C57BL/6J mice can clear the virus during the acute phase and do not develop demyelinating lesions. They instead experience neuronal and neuroparenchymal damage in the hippocampal formation, which results in acute seizures. This pattern of lesion development is most similar to our previous investigations of CC mice during the chronic phase of infection [16], thus C57BL/6J mice were used as a historical comparator in the present study. Previously, we characterized the neurological outcomes produced by long-term TMEV infection in CC mice. We identified a wide spectrum of neurological sequelae and characterized histological profiles of inbred and CC mouse strains at the late chronic phase of infection [16]. Given our previous findings, we hypothesized that the early acute phase of infection would similarly result in diverse clinical signs and histological findings consistent with residual lesions would be observed during the chronic phase [16]. Immune responses, particularly involving microglia or monocytes, and viral clearance in the acute phase of infection drive the long-term presentation of disease phenotypes; this has been well established in studies of TMEV and other viral infection models. Therefore, we extended our examination to include microglial and monocyte involvement by using Iba-1 (ionized calcium-binding adapter molecule 1) immunolabeling, viral load and tropism, using RNA in situ hybridization, and clinical signs and neuropathology during the acute phase. We found that even at the earliest stages of TMEV infection each of the diverse mouse strains included in this study experienced a unique mosaic of responses and, therefore, outcomes. Furthermore, we also identified potential genetic contributors to strain-specific variation in monocyte and microglial activity and viral load. Together, these results provide novel understanding about the early clinical signs of viral-induced neurological diseases, including the underlying neuropathology and microglial/macrophage responses, along with TMEV levels and distribution. We also uncovered genetic determinants which contribute to the long-term consequences of virally induced neurological diseases; these candidate genes may provide mechanistic insights for further study and therapeutic development. 

## 2. Results

### 2.1. Genetic Background, as Represented by Different Mouse Strains, Influenced Clinical Symptom Variability Following TMEV Infection

**Figure 1:**
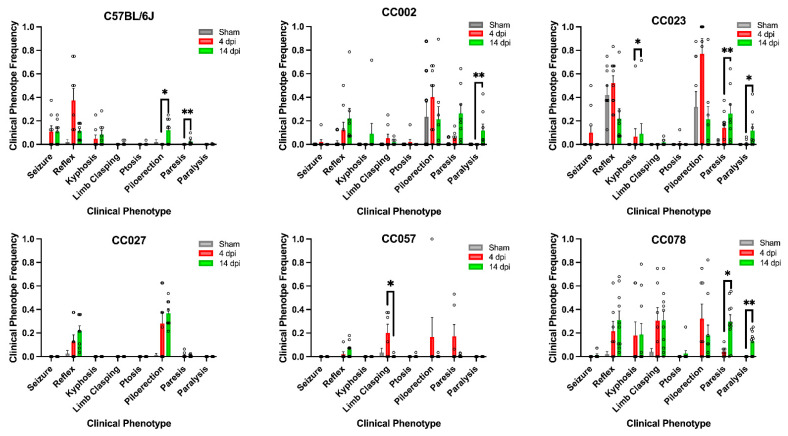
TMEV-induced clinical phenotype frequency varied between mouse strains, infected status, early acute (4 dpi) and late acute (14 dpi) timepoints. Data shown here are the mean ± SEM of the average clinical phenotype frequency for each strain. *p*-values were determined using Wilcoxon rank sum testing; *, *p* < 0.05; **, *p* < 0.01.

To determine which clinical symptoms were significantly affected by TMEV infection, we performed a Wilcoxon rank sum test and found that the average frequency of clinical signs between sham and infected mice varied between strains regardless of dpi (Figure 1). We observed a significant increase in piloerection, a clinical indicator of sickness, in CC002 (*p* = 0.0299), CC023 (*p* = 0.0027), CC027 (*p* = 0.008), and CC078 (*p* = 0.009) in infected mice compared with shams. The average frequency of seizure behavior was significantly increased in infected mice compared with shams for C57BL/6 (*p* = 0.0013) and CC023 (*p* = 0.045). There were no significant differences in seizure frequency between the other strains. All strains exhibited significant differences in frequency of delayed righting reflex (C57BL/6, *p* ≤ 0.0001; CC002, *p* = 0.0054; CC023, *p* = 0.029; CC027, *p* = 0.0034; CC057, *p* = 0.039; CC078, *p* = 0.002). Another common feature across strains and timepoints was an increase in limb paresis. All infected mice showed an increase in paresis (C57BL/6, *p* = 0.0135; CC002, *p* ≤ 0.0001; CC023, *p* ≤ 0.0001; CC027, *p* = 0.0086; CC057, *p* = 0.0135; CC078, *p* = 0.002). However, only infected mice from strains CC002 (*p* = 0.0136), CC023 (*p* = 0.005), and CC078 (*p* = 0.014) showed an increase in limb paralysis compared to shams. We also examined kyphosis in these mice and found a significant increase in frequency of hunching and kyphosis for C57BL/6 (*p* = 0.0065), CC023 (*p* = 0.0108), and CC078 (*p* = 0.0264) in infected mice compared to shams. Lastly, we found that infected mice from strains CC002 (*p* = 0.0271) and CC078 (*p* = 0.0192) had significant differences in limb-clasping behavior post-TMEV infection. Limb clasping for the other strains did not significantly differ from shams. Summary statistics can be found in Supplementary Tables S2–S7.

### 2.2. Female and Male Mice Did Not Exhibit Significant Differences in TMEV-Induced Clinical Signs during the Acute Phase of Viral Infection

To determine whether there were sex differences in TMEV-induced clinical signs, we applied the Wilcoxon rank sum test to the infected mice. There were no significant differences between sexes in any strain for any clinical sign except hunching/kyphosis in C57BL/6, where there was a statistical difference (*p* = 0.0092) (Supplemental Figure S1). Summary statistics can be found in Supplemental Tables (Supplemental Tables S8–S13).

### 2.3. Frequencies of TMEV-Induced Phenotypes Varied by Strain between 4 dpi and 14 dpi

We examined the changes in average phenotype frequencies from 4 to 14 dpi, to determine which clinical symptoms were significantly different for each strain between the early and later acute phases of infection (Figure 1). We found that CC023 mice had a significant difference in frequency of kyphosis observed at the 4 vs. 14 dpi timepoints (*p* = 0.0179). We found a significant difference in frequency of piloerection between the two timepoints for C57BL/6 (*p* = 0.0126), and in limb-clasping frequency for CC057 (*p* = 0.0204). We also observed significant differences between the frequency of paresis for C57BL/6 (*p* = 0.0044), CC023 (*p* = 0.0055), and CC078 (*p* = 0.0213). Finally, we detected a significant difference in phenotype frequency for paralysis for CC002 (*p* = 0.0046), CC023 (0.0102), and CC078 (0.0087). There were no significant differences in the frequency of seizures or delayed righting reflex between 4 and 14 dpi for any of the strains. 

### 2.4. Histologic Lesions of the Brain, Iba-1 Immunolabeling, and TMEV mRNA Expression in the Hippocampal Formation Demonstrated the Influences of Genetic Background

Across all analyzed CC strains, at 4 and 14 dpi, lesions were more pronounced in the hippocampal formation than in other locations. Additional affected areas included the striatum and thalamus. As expected [16], no lesions were observed in the cerebellum or brainstem (Level D). Within the hippocampal formation, fields CA1 and CA2 were consistently affected across all CC strains and timepoints (Table 1). The dentate gyrus was spared in all strains except CC023. 

**Figure 2:**
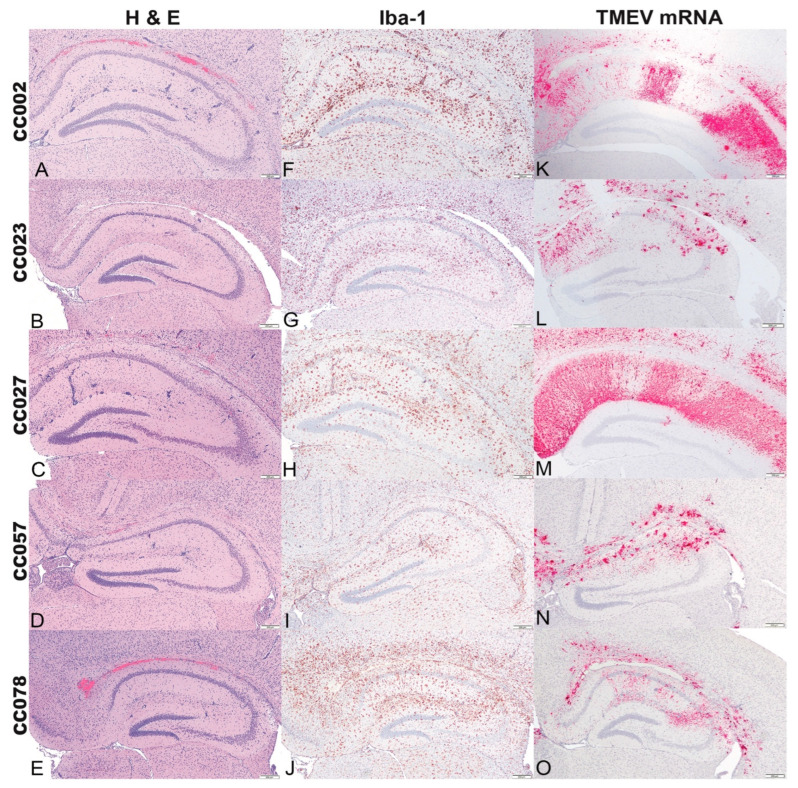
Cross sections of the hippocampal formation at level B of CC mice infected with Theiler’s murine encephalomyelitis virus (TMEV) and euthanized at 4 dpi. (**A**–**E**) (Hematoxylin and eosin stain) Mild multifocal neuronal necrosis of CA1 and CA2 pyramidal layers with multifocal glial aggregates mostly centered around vessels (perivascular cuffing), predominantly in the stratum radiatum, stratum lacunosum moleculare. A linear area of hemorrhage in the alveus and hippocampal commissure was observed in A and E, interpreted as secondary to intracerebral TMEV inoculation. (**F**–**J**) (Iba-1 immunolabeling) All infected CC mice demonstrated increased numbers of Iba-1 positive microglia/macrophages in the hippocampal formation, most pronounced in the CC078 strain. (**K**–**O**) (TMEV RNA in situ hybridization) In all infected CC mice, mRNA expression was broadly distributed throughout the hippocampal formation. CC002 and CC027 strains had a radiating pattern of mRNA expression while CC023, CC057, and CC078 showed clustered patterns. Bar = 200 μm.

**Figure 3:**
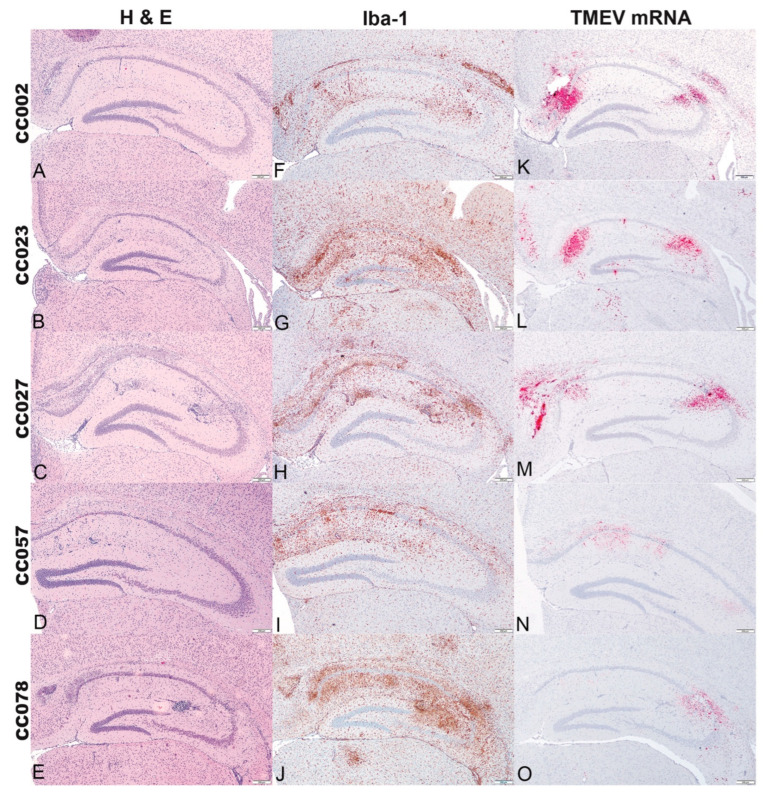
Cross sections of the hippocampal formation at level B of CC mice infected with Theiler’s murine encephalomyelitis virus (TMEV) and euthanized at 14 dpi. (**A**–**E**) (Hematoxylin and eosin stain) Locally extensive areas of neuronal necrosis and loss were observed in CA1 and CA2 pyramidal layers with granular mineralization of the stratum oriens above CA1. The stratum radiatum and stratum lacunosum moleculare exhibited moderate to marked gliosis and perivascular cuffing. (**F**–**J**) (Iba-1 immunolabeling) Increased numbers of Iba-1 positive microglia/macrophages were widely distributed throughout the hippocampal formation. In the CC023 strain, the gliosis encompassed the dentate gyrus. (**K**–**O**) (TMEV RNA in situ hybridization) TMEV mRNA expression was confined to the medial area of fields CA1 and CA2. In the CC023 strain, TMEV mRNA expression was observed in the dentate gyrus and underlying thalamus. Bar = 200 μm.

At 4 dpi, the lesions consisted mainly of multifocal neuronal necrosis of CA1 and CA2 pyramidal layers with neuroparenchymal glial aggregates and perivascular cuffing in the stratum radiatum, stratum lacunosum moleculare, and stratum oriens, with occasional punctate mineralization (Figure 2A–E). Microglial/macrophage reactivity was broadly distributed in the hippocampal formation (Figure 2F–J) and coincided with TMEV mRNA expression (Figure 2K–O). At 14 dpi, the main histological findings included locally extensive neuronal necrosis and loss in the pyramidal layers of CA1 and CA2 (Figure 3A–E). Within the stratum oriens there was extensive mineralization in fields CA1 and CA2, as compared to 4 dpi. Microglial/macrophage reactivity was more pronounced than at 4 dpi (Figure 3F–J), and neuroparenchymal and perivascular microglia/macrophages were observed. Relative to 4 dpi, TMEV mRNA expression was more limited to the medial area of field CA1 and sparsely distributed throughout field CA2 (Figure 3K–O). For comparison, data for the the histologic lesions, Iba-1 immunolabeling, and TMEV mRNA expression in the hippocampus of C57BL/6J mice, at both 4 dpi and 14 dpi, can be found in Supplemental Figure S2. 

### 2.5. Hippocampal Microglial/Macrophage Reactivity Varied over Time and by Genetic Background

**Figure 4:**
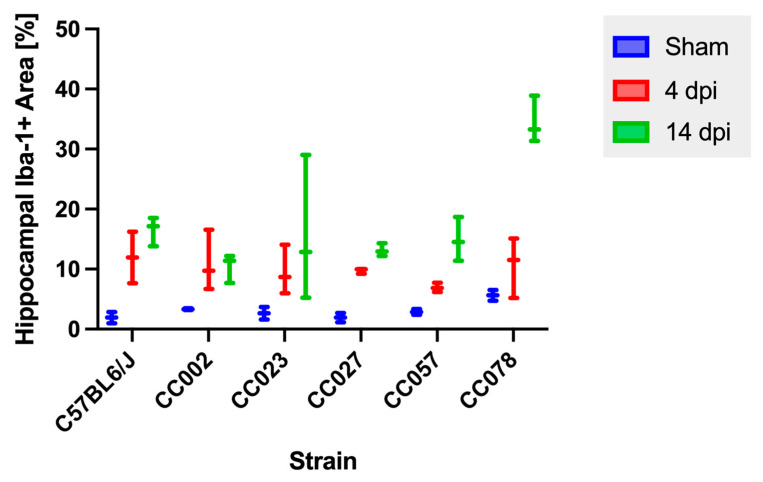
Quantification of the Iba-1 immunopositive area within the hippocampal formation following intracranial inoculation with Theiler’s murine encephalomyelitis virus. No mice showed a significant change in the Iba-1+ area between 4 dpi and 14 dpi. *p* values were determined using the Wilcoxon rank sum tests.

We measured the percentage of Iba-1+ immunopositive areas within the hippocampal formation and found that there were significant differences between sham and infected animals at both 4 dpi (*p* = 0.0009) and 14 dpi (*p* = 0.0004) (Figure 4). To further examine the differences between strains and timepoints, we compared the percentage of Iba-1+ area at 4 dpi and 14 dpi for each strain. We did not detect statistically significant differences in Iba-1+ areas between 4 and 14 dpi within strains. 

### 2.6. TMEV mRNA Expression Decreased between 4 dpi and 14 dpi in All Strains

**Figure 5:**
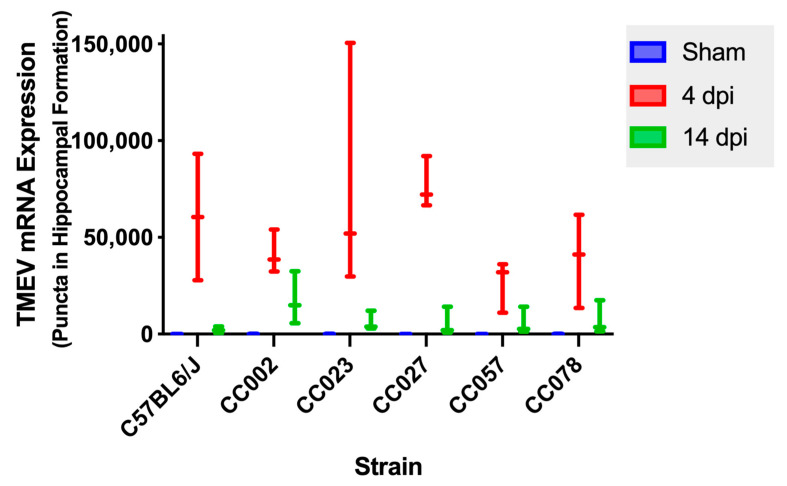
Theiler’s murine encephalomyelitis virus mRNA expression within the hippocampal formation varied by strain and time point. TMEV mRNA expression differed between sham and infected mice and generally decreased between the 4 dpi and 14 dpi timepoints. *p*-values were determined using the Wilcoxon rank sum tests.

We measured TMEV mRNA expression in the hippocampal formation at 4 dpi and 14 dpi (Figure 5). We compared TMEV mRNA expression between 4 dpi and 14 dpi for each strain and observed a trend towards significance for CC023 (*p* = 0.0809) and CC027 (*p* = 0.0809). We did not detect a significant difference between 4 dpi and 14 dpi for the other strains (C57BL6/J, *p* = 0.1489; CC002, *p* = 0.1904; CC057, *p* = 0.1904; CC078, *p* = 0.1904). However, there was a general trend in decreasing TMEV mRNA expression between 4 dpi and 14 dpi in all strains. 

### 2.7. Microglial/Macrophage Reactivity Was Correlated with Viral Load at 4 dpi

We looked for possible correlations between microglial/macrophage reactivity and viral load at 4 dpi and 14 dpi. We found that there was a moderate positive correlation between high microglial/macrophage reactivity and high viral load at 4 dpi (*p* = 0.003, r = 0.69). This positive correlation was statistically significant at 14 dpi (*p* = 0.0065, r = 0.54). 

### 2.8. Several Loci of Interest Were Associated with Lesion Distribution and Microglial/Macrophage Reactivity

**Figure 6:**
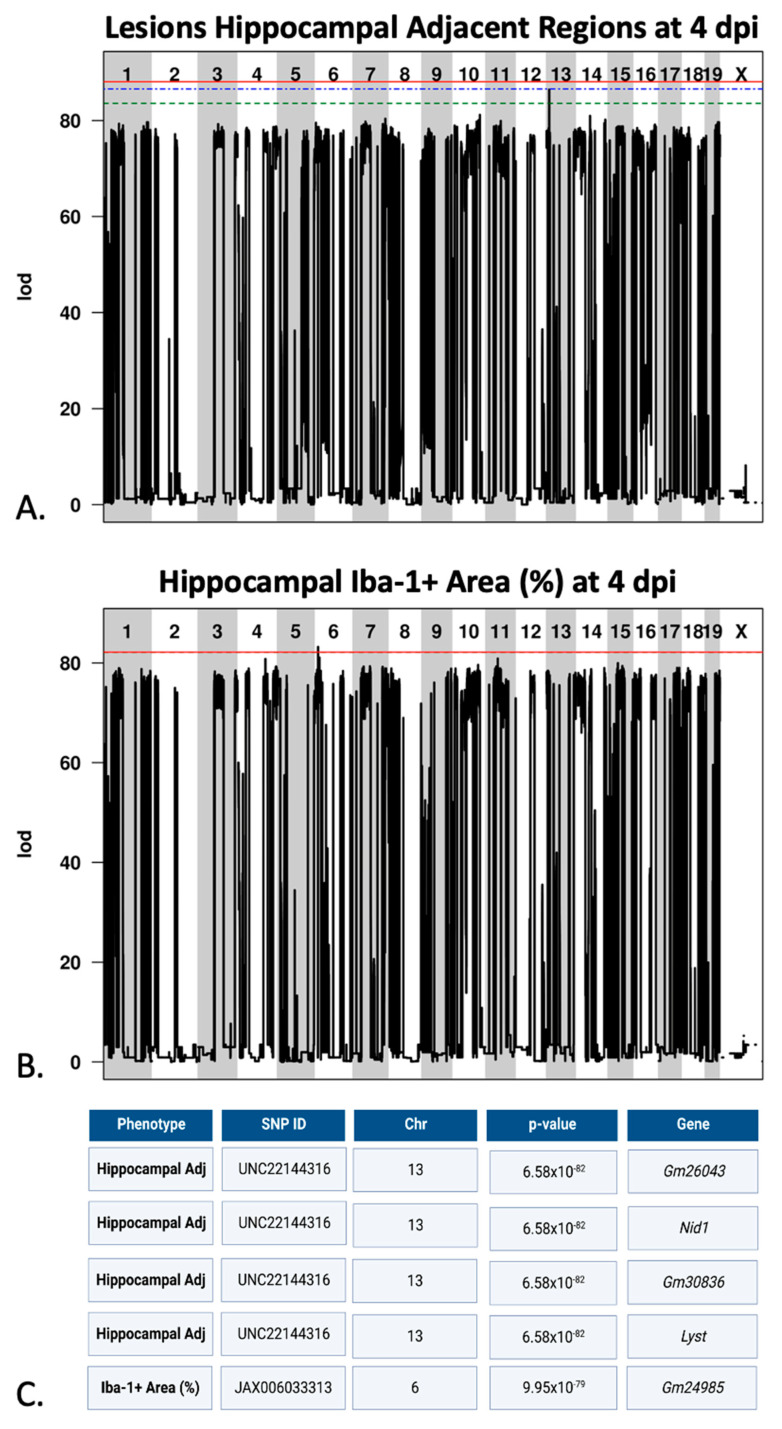
Significant quantitative trait loci (QTL) associated with lesion frequency and microglial/macrophage reactivity following Theiler’s murine encephalomyelitis virus infection. (**A**) QTL analysis using gQTL revealed a region on mouse chromosome 13 which was significantly associated with lesion frequency in hippocampal adjacent regions at 4 dpi. (**B**) QTL analyses revealed a statistically significant region on chromosome 6 associated with microglial/macrophage reactivity at 4 dpi. (**C**) The association between the single nucleotide polymorphism (SNP) UNC22144316 on chromosome 13 and lesion frequency at 4 dpi was statistically significant (*p* = 6.58 × 10^−81^). Within that QTL interval were four genes of interest: *Gm26043*, *Lyst*, *Gm30836*, and *Nid.* The SNP JAX006033313 on chromosome 6 was significantly associated (*p* = 9.96× 10^−79^) with the level of microglial/macrophage reactivity at 4 dpi. One predicted gene, *Gm24985*, within this interval was just adjacent to another gene of interest: *Tmem106b*.

We next performed genetic analyses to identify genetic loci contributing to variation in the TMEV-induced phenotypes we observed. For this, we used gQTL, a web-based software program designed specifically for use with the many diverse genomes represented by CC strains (see Materials and Methods). Quantitative values included numbers of mice with lesions in a given region for each strain, or relative area occupied by Iba1+ microglia for each strain (Iba1+ indicates the presence of activated microglia/macrophages). We identified several genomic regions associated with lesion location and microglial/macrophage reactivity at 4 dpi (Figure 6). We detected statistically significant associations between the frequency of lesions at 4 dpi in the hippocampal adjacent region and a locus on chromosome 13 between base positions 13,472,643–13,654,592 (mouse genome build 38, mm10). This region was also identified in previous investigations of the chronic (90 dpi) phase of infection [16]. Of the four loci located in this region, lysosomal trafficking regulator (*Lyst*) and nidogen 1 (*Nid1*) may be candidates for further study due to their known functional relevance [17]. In addition to the associations found for lesion frequency and distribution, we identified an association with Iba-1 immunopositive area of the hippocampal formation at 4 dpi and a locus located on chromosome 6 between base positions 13,096,370–13,143,710 (mouse genome build 38, mm10). *Tmem106b* is located immediately adjacent to this QTL on chromosome 6, at positions 13,069,759–13,089,269, and may be of particular interest due to its roles in similar conditions such as hypomyelinating leukodystrophy [18,19]. We found no significant QTL associated with viral load at 4 dpi, 14 dpi, or rate of viral clearance. 

## 3. Discussion

Viral-induced neuroinflammation is a possible driver for many neurodegenerative diseases [20]. It is critically important to determine relationships, particularly those involving early host responses, between viral infection and the development of neurological disease, which can ultimately lead to long term neuropathology. Early response and viral clearance are important in determining long-term disease processes [20]; these elements vary between individuals, leading to different prognoses. Among the many factors which determine susceptibility, viral persistence, and clinical outcomes, host genetics have a critical influence [21]. For this reason, viral infection in different populations can have very divergent outcomes. We modeled this population diversity by using five CC strains to characterize neuropathologic changes occurring at two time points of the acute phase of TMEV infection. We previously evaluated these same CC strains during the chronic phase of TMEV infection [16]. As a result, we have longitudinal profiles for each of these strains, valuable for model development and/or identification of shared and unique mechanisms underlying neuropathogenesis. Accordingly, the work described in this study represents the first experimental investigations to characterize TMEV load and tropism in the hippocampus of genetically diverse mouse strains. We found that viral load correlated with macrophage/microglial reactivity, regardless of genetic background. These results form the basis for future investigations examining the effect of host genetics on neurological and immune responses to viral infection, and investigating how early host responses set the stage for variation in long-term disease outcomes. 

We observed strain-specific variation in clinical signs during the acute phase of infection, similar to our previous findings for the chronic phase of TMEV infection in CC mice [16,22,23]. Additionally, we found that clinical signs, excluding those associated with sickness, tended to worsen between 4 dpi and 14 dpi. Strain-specific variation in clinical signs was appreciable as early as 4 dpi, and several strains (CC002, CC023 and CC078) displayed significantly worsening motor deficits (e.g., paresis and paralysis) between 4 and 14 dpi (Figure 1). CC002, CC023, and CC078 continued to exhibit worsening motor phenotypes during the chronic phase of infection, based on our prior findings, while the phenotypes of CC057 improved slightly, and CC027 remained relatively unaffected [16]. This longitudinal data reveals novel clues about the phenotypic manifestations of different neuropathological states at different phases of the infection.

TMEV has specific tropism for the CA1 and CA2 pyramidal layers of the hippocampus, periventricular thalamic nuclei, septal nuclei, and piriform, parietal, and entorhinal cortices ([16,24,25,26]). In our acute phase study, we observed lesions in the striatum, thalamus, and hippocampal formation. However, across these brain regions, the hippocampal formation was preferentially and consistently affected across the five CC strains. Like C57BL/6J, TMEV-infected CC mice had marked neuronal necrosis and gliosis in fields CA1 and CA2 [15,25]. These histopathological features mirror descriptions of lesions found in epileptic foci in the brains of human patients with temporal lobe epilepsy [27,28,29]. Interestingly, we did not observe seizures in all CC strains, possibly due to individual strain resistance to TMEV’s neuropathological damage. Specifically, it appeared the CC027 mice were able to sustain lesions without showing severe clinical signs, compared with CC057 and CC078 that showed lesions in similar locations. Unlike SJL/J mice, which develop neuronal necrosis and microglial proliferation and perivascular mononuclear cuffing in the thalamus, brainstem, and cerebellum [30,31], none of the CC strains in this study developed similar lesions in the brainstem or cerebellum. 

Immune system activation within the central nervous system (CNS) can be elicited by infection, namely by neurotropic viruses, resulting in peripheral monocyte infiltration into the CNS and the subsequent activation of resident microglial cells [32,33]. Monocyte infiltration and activation of microglia are hallmarks of CNS inflammation, including viral infection [34]. However, the role of these cells in viral clearance and immunopathology has not been well defined, partly due to challenges in differentiating invading monocytes from activated microglia in the brain, and a lack of selective tools to manipulate these two types of macrophages [34]. However, according to recent suggestions, differences in neuroinflammatory responses of monocytes and microglia may play an important role in viral clearance, CNS pathology, and disease development [34,35]. In several viral brain infections, activated microglia appear to be involved in the inhibition of viral replication and in neurotoxicity, indicating the dual nature of microglia; they contribute to the defense of the CNS but may also be responsible for CNS damage [32]. Specifically, microglia have been shown to play an integral role in influencing TMEV-induced responses and provide a protective or immunoregulatory function that may affect other effector cell populations like inflammatory monocytes [34,35]. 

In the present study, we found a positive correlation between microglial/macrophage reactivity and viral load. This was not surprising: TMEV mRNA has been shown to be expressed in microglia [36]. In our study it appeared that TMEV mRNA expression was localized in neurons and microglia/macrophages. However, colocalization studies will be necessary to determine any strain-specific variation in the cell types and TMEV mRNA expression. We also observed a significant increase in microglial/macrophage reactivity in infected mice, with significant changes in three strains (CC027, CC057, and CC078) between the early (4 dpi) and late (14 dpi) acute phases of TMEV infection. Interestingly, these strains experienced very different clinical signs, ranging from mild (CC027) to moderately severe (CC057 and CC078) profiles. This indicated that macrophage/microglial reactivity did not have a strong effect on the type, severity, improvement, or worsening of clinical signs during the acute phase. We previously showed that TMEV RNA levels did not correlate with clinical signs or severity [37,38]; this further underscores the potentially crucial roles for specific cell types in influencing clinical manifestations of neuropathology.

In the current study we included C57BL/6J as a historical comparator. The almost total lack of TMEV RNA by 14 dpi (Figure 5) concurred with differences in microglial activation shown in Supplementary Figure S2. Previous investigations into the role of microglia in C57BL/6J mice showed that PLX5622-mediated microglial depletion resulted in fatal viral encephalitis [39]. Interestingly, paralysis was also observed in these mice in addition to seizures. Depletion of microglia worsened seizure activity and also increased hippocampal damage in C57BL/6J mice [34], demonstrating that microglia, and probably CNS-infiltrating macrophages, have distinct roles in antiviral immune response and development of clinical signs such as seizure and paralysis [39]. 

The damage caused by TMEV infection, whether directly from the virus itself or due to the antiviral immune response, did not result in the same lesion profiles across all strains, although there were common characteristics among the lesions across all strains. Given the genetic diversity of the strains used in this study, and our previous identification of QTL associated with lesion burden [16], we hypothesized the existence of one or more genetic contributors to lesion prevalence and location. Our QTL search revealed a significant region of interest associated with frequency of lesions found in the hippocampal adjacent areas during the early acute phase of infection (4 dpi). This region harbored relevant genes of potential interest, and was the same region previously identified in our investigation of the chronic phase of infection (90 dpi), also in relation to hippocampal adjacent regions [16]. Possible reasons that this QTL was not identified as significant at 14 dpi could be the lack of variation in the presence of lesions in that area, and the fact that the lesions were at their most extensive at 4 dpi. Furthermore, compared with 14 dpi, the residual damage at 90 dpi [16] caused more neuropathological changes outside the hippocampus and affected hippocampal adjacent regions (alveus and dorsal hippocampal commissure). The region of the significant QTL harbored four genes, including two “predicted genes” and two known genes which we considered of interest due to their potential relevance. These genes were lysosomal trafficking regulator (*Lyst*) and nidogen 1 (*Nid1*), both of which we previously described in relation to lesion frequency during the chronic phase of infection [16]. Briefly, these genes have important roles in viral-induced pathology. *Lyst* has been shown to play an immunoregulatory role in the proinflammatory responses of toll-like receptors (TLRs), namely of TLR3 and TLR4 [17], and has been associated with hemophagocytic lymphohistiocytosis following Epstein–Barr infection in a human patient [40]. Next, cytomegalovirus infection in humans has been shown to cause downregulation of nidogen 1 (*Nid1*) and to degrade the protein it encodes, thus impacting vascular wall integrity and allowing further viral spread [41]. Therefore, *Lyst* and *Nid1* are likely contributors to TMEV-induced inflammation and lesion responses, and should be investigated further in the context of TMEV and the CC mouse resource. 

Our QTL search also revealed a significant association at 4 dpi between the Iba-1 immunopositive area of the hippocampal formation and a very small region on chromosome 6 containing one predicted gene. However, we determined the gene *Tmem106b* to be of particular interest and a likely reason for the significant association, due to its position nearly overlapping the significant QTL region. *Tmem106b* encodes a protein (TMEM106B) that is involved in lysosome function and has been implicated in neurodegenerative disorders [18,42]. Additionally, a recent study implicated the lysosomal protein TMEM106B in viral entry for SARS-CoV-2 infection, possibly by promoting endosomal acidification to facilitate the delivery of the SARS-CoV-2 genome into the cytoplasm [43]. TMEM106B has also been implicated in frontotemporal dementia in humans, which has been recently recognized as a disease with a common pathogenic background with ALS [44]. A role for *Tmem106b* in TMEV neuropathogenesis seems likely, and therefore this gene and the protein it encodes will be targets for future study.

Given the longstanding value of TMEV infection as a model for studying neuropathological outcomes to viral infections, it is critical to understand how and why individuals from different genetic backgrounds can experience different outcomes. The current study has set the stage for defining the role(s) of infiltrating monocytes and microglia on clinical signs and CNS damage. Ultimately, our findings can inform the development of novel biomarkers for virus-induced neurological conditions, by demonstrating how differences in viral clearance and neuroinflammation shape the physiological manifestations of disease.

## 4. Materials and Methods

### 4.1. Mouse Care 

All animal care protocols were in accordance with NIH Guidelines for Care and Use of Laboratory Animals and were approved by the Texas A&M University Laboratory Animal Care and Use Committee (AUP 2020-0065). Breeding of all C57BL/6J and CC mice was performed in-house at Texas A&M University.

The mice were maintained in an AAALAC approved facility under 14-h light and 10-h dark cycle with ad libitum food and water. Ninety-six mice between three and four weeks of age, including females and males from inbred strain C57BL/6J (used as a historical comparator), and five CC strains (CC002, CC023, CC027, CC057, and CC078) were used. At each timepoint, six to ten mice per strain (Supplementary Table S1), were anesthetized by isoflurane inhalation (MWU, Meridian, ID, USA) and intracerebrally injected into the right mid-parietal cortex (approximately 1.5 mm ventral) with 5.0 × 10^4^ plaque-forming units (PFU) of the BeAn strain of TMEV (American Type Culture Collection [ATCC] VR 995, Manassas, VA, USA) in 20 μL of phosphate buffered saline (PBS). Sham infected mice (n = 84, including females and males representing the same mouse strains, six to ten mice per strain and timepoint) were anesthetized and intracerebrally injected with PBS only. Mice were randomized into treatment groups, and were housed four or five to a cage, observed and weighed daily to monitor general health. Measures were taken to minimize mouse discomfort and stress, including the provision of softened food pellets if necessary. Mice that lost more than 20% of their pre-infection body weight prior to the 4 or 14 days post infection (dpi) endpoint were euthanized and excluded from the study.

### 4.2. Evaluation of Clinical Signs

All mice were evaluated using established methods for evaluating TMEV-induced clinical signs [16,22,23,37,38]. Mice were assessed for clinical signs twice per day, at the same times of day, from 0–14 dpi. The clinical signs examined included delayed righting reflex, paresis, paralysis, seizures, limb clasping, kyphosis, ptosis, and piloerection. The clinical phenotype frequency at 4 dpi and 14 dpi was calculated as the number of observations for each clinical sign for each mouse strain, divided by the total number of observations by that time point [16,22]. 

Briefly, delayed righting reflex was assessed by turning each mouse on its back on a flat surface and measuring the time taken to right itself to a prone position with all four paws underneath it, for at least two trials. A delay in righting reflex is indicative of vestibular pathway disfunction, spinal interneurons, proprioceptive afferents, and motor neurons [45,46]. Paresis and paralysis were evaluated by allowing the mice to walk on a flat surface, and by placing each mouse on a metal grate and inverting the grate [47]. Mice were allowed to navigate the grate and each of the limbs were scored as described in Lawley et al., 2021 [16,22,23,37]. Paresis was recorded for each limb that retained limited movement. Paralysis was recorded if a mouse was unable to move a limb on a flat surface or if a limb was unable to grip or remain on the grate. We evaluated the presence or absence of seizures and limb clasping [48]. Kyphosis (hunched posturing), which has been associated with ALS models [49], was also documented. We also recorded clinical signs associated with sickness behaviors (i.e., ptosis and piloerection) as described by Lawley et al., 2021 [50,51,52]. 

### 4.3. Euthanasia and Tissue Collection

Mice were euthanized at 4 or 14 dpi via intraperitoneal (i.p.) injection of a lethal dose of Beuthanasia -D Special 150 mg/kg (Schering-Plough Animal Health) as previously described [25]. Mice were transcardially perfused through the left ventricle with 10 mL of ice-cold phosphate buffered saline. Following perfusion, necropsy was performed on each mouse, and the left hemisphere of the brain was collected and fixed for 48 h in 10% formalin.

### 4.4. Histology

Coronal sections of the mice brains were collected at four different levels as described in Lawley et al., 2021: level A (frontoparietal cortex, septal nuclei and caudate-putamen, nucleus accumbens), level B (frontoparietal cortex, hippocampus [CA regions—1, 2, 3, dentate gyrus], and thalamus), level C (occipital cortex and rostral colliculi of the midbrain), and level D (cerebellum, cerebellar peduncles and pons). All sections were routinely processed with hematoxylin and eosin (H&E) stain. All slides were reviewed by a board-certified veterinary pathologist, blinded to slide identity. Representative images were acquired using an Olympus BX43-F microscope at 40× magnification with a DP73 camera, ND filters, and CellSens Standard Software.

### 4.5. Iba-1 Immunohistochemistry of the Hippocampal Formation

One sham mouse and three infected mice for each strain were selected at 4 dpi and 14 dpi timepoints for Iba-1 immunohistochemistry. Iba-1 is expressed by microglia and macrophages and is involved in early phagocytosis; it is commonly used as a pan-macrophage marker [13,53]. The immunohistochemistry protocol was optimized and standardized using an autostainer. The following protocol was used: Antigen retrieval was performed with concentrated Antigen Retrieval Citra Solution (BioGenex HK986-9K, Fremont, CA, USA) for 15 min at 110 °C. Endogenous peroxidase activity was blocked using 3% hydrogen peroxide (Fisher H324-500, Hampton, NH, USA) for 5 min. Universal Blocking Reagent 10X (Power Block) (BioGenex HK085-5K, Fremont, CA, USA) was applied for 5 min. Sections were then incubated for 60 min with rabbit antibody against Iba-1 (019-19741, Wako Chemicals, Richmond, VA, USA [1:8000]) followed by a 10-min incubation with biotinylated Goat Anti-Rabbit (Vector BA-1000, 1:100). Detection was performed with 4+ Streptavidin HRP Label (Bio Care Medical, Ap604H, Pacheco, CA, USA) for 10 min and Beazoid DBA Chromagen Kit (Bio Care Medical, BDB2004L, Pacheco, CA, USA) for 12 min. 

### 4.6. RNA In Situ Hybridization of the Hippocampal Formation

The same mice used for Iba-1 immunohistochemistry were selected for RNA in situ hybridization. The formalin-fixed paraffin-embedded brain sections were cut to 3 uM sections, mounted on charged slides, incubated in an oven for 1 h at 60 °C and RNAscope 2.5 HD red assay (Advanced Cell Diagnostics, 322360, Newark, CA, USA) was performed according to the manufacturer’s protocol and guidelines (RNAscope; Advanced Cell Diagnostics, Hayward, CA, USA) with minor modifications. Modifications of the protocol included a reduced boiling time in the target retrieval buffer (15 min total) and reducing the Protease Plus incubation to 15 min. The sample preparation and pretreatment included deparaffinization in xylene, followed by dehydration with 100% ethanol and distilled water. Brain sections were air-dried and incubated with hydrogen peroxide for 10 min at room temperature then rinsed with distilled water. Samples were then boiled for 15 min at 98–102 °C in a pretreatment solution (Advanced Cell Diagnostics, 322000, Newark, CA, USA) and washed in distilled water followed by 100% ethanol. Slides were air-dried and incubated with RNAscope protease reagent for 15 min at 40 °C in a HybEZ hybridization oven (Advanced Cell Diagnostics, 310010, Newark, CA, USA). After rinsing with distilled water, brain sections were hybridized with TMEV or control probes at 40 °C for 2 h in a hybridization oven. We used a mouse RNAscope Probe-Mm Ppib positive control probe (Advanced Cell Diagnostics, 313911, Newark, CA, USA) and the bacterial RNAscope DapB probe (Advanced Cell Diagnostics, 310043, Newark, CA, USA) as a negative control. Our target probe was specifically for detection of TMEV (Advanced Cell Diagnostics, 532991). Hybridization with AMP1–AMP6 reagents was performed as follows: AMP1 (40 °C for 30 min), AMP2 (40 °C for 15 min), AMP3 (40 °C for 30 min), AMP4 (40 °C for 15 min), AMP5 (room temperature for 1 h), and AMP6 (room temperature for 15 min). Between each hybridization step, slides were washed with wash buffer for 5 min three times. The signal amplification was performed with a horseradish peroxidase-based system followed by chromogenic detection with 3,3′-diaminobenzidine. Slides were counterstained with Gill’s hematoxylin and mounted with a xylene-based non-aqueous mounting media. 

### 4.7. Digital Analysis of Iba-1 Immunohistochemistry and RNA In Situ Hybridization

Slides were scanned at 40× magnification using the Pannoramic Scan II by 3DHistec, and images were analyzed using Visiopharm Software (Version 2018.4, Hoersholm, Denmark) [54,55]. Briefly, the hippocampal formation was outlined manually as the region of interest (ROI), specifically, field CA1, field CA2 and field CA3 (including the pyramidal layers, the suprajacent stratum oriens, the subjacent stratum radiatum, and stratum lacunosum moleculare), and the dentate gyrus. For both the Iba-1 and RNA in situ hybridization, a custom Visiopharm APP was developed to assess the Iba-1 immunopositive area of the hippocampal formation, as well as the signal count of TMEV mRNA expression. For Iba-1, the protocol detected the area of Iba-1 immunopositivity and determined the percentage of the ROI immunopositive for Iba-1. For RNA in situ hybridization, the punctate dots or clusters within the ROI were enumerated, each of which represented a single mRNA copy,. Both measures were then subjected to statistical analysis. 

### 4.8. Statistical Analysis

In analyzing the phenotype data for each strain of mouse (C57BL/6J, CC002, CC023, CC027, CC057, CC078) we calculated descriptive statistics including mean and standard deviation for sex (male versus female), infection status (infected vs. sham), and dpi (4 vs. 14 dpi) for each clinical phenotype (seizure, paresis, etc.). There were no assumptions of normality, so a nonparametric method was used to test significance. Within each strain, Wilcoxon rank sum testing was applied to determine whether there was a significant difference between sex, dpi, or infection status for each measurement of clinical phenotype. For sex and dpi, sham mice were removed. The Wilcoxon rank sum test was also used to determine whether there was a significant difference between sham and infected mice in percentage of Iba-1+ immunopositive area within the hippocampal formation at 4 and 14 dpi. Shams were then removed and using the same method we compared 4 dpi and 14 dpi for each strain of mouse. Spearman correlation was used determine any linear relationship between microglial/macrophage reactivity and levels of TMEV mRNA expression. A *p*-value of ≤ 0.05was considered statistically significant. All analyses were performed using Statistical Analysis System (SAS) software, version 9.4 (SAS Institute, Cary, NC, USA).

### 4.9. Genetic Association Analysis

We calculated the frequency of lesion presence for each CC strain at 4 dpi and 14 dpi according to the number of mice with a lesion observed in a given brain region, divided by the total number of mice analyzed for that strain. To identify genomic regions associated with the calculated lesion frequency, we used the gQTL online software platform [56] to analyze the genomes of CC002, CC023, CC027, CC057 and CC078. Significance thresholds were determined using 1000 permutations and *p*-value of less than 0.05. To determine genomic regions associated with macrophage/microglial reactivity in the hippocampal formation, we used the percentage of immunopositive area of Iba-1+ macrophages at 4 and 14 dpi. Additionally, we identified QTL associated with viral load (as measured by enumeration of TMEV mRNA puncta within the hippocampal formation) at both timepoints, and the differences between timepoints were examined. The values used for gQTL analysis were strain averages, with values from sham-infected mice subtracted from those of infected mice for each strain. By subtracting the sham values, we removed strain-specific background variation unrelated to TMEV infection. 

## Figures and Tables

**Table 1:** Frequency of TMEV-infected mice with lesions in the brain regions shown at 4 dpi and 14 dpi. Color gradient reflects relative frequency values, from 0 (green) to 1 (red). * Field includes stratum oriens, pyramidal layer, stratum radiatum, and stratum lacunosum-moleculaire. † Adj: adjacent regions of the hippocampus (dorsal hippocampal commissure and alveus).

Mouse Strain		Hippocampal Formation			^†^ Hippo. Adj.	Striatum			Thalamus
		* Field CA1	* Field CA2	* Field CA3		Caudate-Putamen	Septal Nuclei	Nucleus Accumbens	
4 dpi–early acute	C57BL/6J	0.86	0.86	0.29	0	0.29	0.29	0	0.29
	CC002	1	1	0.13	0.25	0.50	0.625	0.25	0.50
	CC023	0.9	0.40	0.10	0.50	0.20	0.20	0.20	0.30
	CC027	1	0.71	0	0	0.14	0.14	0	0
	CC057	1	0.29	0	0.14	0.43	0.43	0	0.29
	CC078	0.63	0.50	0	0	0.25	0.38	0	0.13
14 dpi–late acute	C57BL/6J	0.86	0.86	0.38	0	0.13	0.38	0	0.63
	CC002	1	0.75	0	0.13	0.13	0.63	0	0.50
	CC023	0.63	0.38	0	0	0	0.25	0.13	0.63
	CC027	1	0.88	0	0	0.13	0	0	0.38
	CC057	0.89	0.67	0	0.22	0	0	0	0.56
	CC078	1	0.36	0	0.09	0.55	0.36	0	0.36

## Data Availability

Not applicable.

## Supplementary Materials

The following supporting information can be downloaded at: https://www.mdpi.com/article/10.3390/ijms231810482/s1.

## References

[1] Donati D. (2020). Viral infections and multiple sclerosis. Drug Discov. Today Dis. Models.

[2] Xue Y.C., Feuer R., Cashman N., Luo H. (2018). Enteroviral Infection: The Forgotten Link to Amyotrophic Lateral Sclerosis?. Front. Mol. Neurosci..

[3] Jang H., Boltz D., McClaren J., Pani A.K., Smeyne M., Korff A., Webster R., Smeyne R.J. (2012). Inflammatory effects of highly pathogenic H5N1 influenza virus infection in the CNS of mice. J. Neurosci..

[4] Itzhaki R.F., Wozniak M.A. (2006). Herpes simplex virus type 1, apolipoprotein E, and cholesterol: A dangerous liaison in Alzheimer′s disease and other disorders. Prog. Lipid Res..

[5] Jacobs B.C., Rothbarth P.H., van der Meche F.G., Herbrink P., Schmitz P.I., de Klerk M.A., van Doorn P.A. (1998). The spectrum of antecedent infections in Guillain-Barre syndrome: A case-control study. Neurology.

[6] Karatas H., Gurer G., Pinar A., Soylemezoglu F., Tezel G.G., Hascelik G., Akalan N., Tuncer S., Ciger A., Saygi S. (2008). Investigation of HSV-1, HSV-2, CMV, HHV-6 and HHV-8 DNA by real-time PCR in surgical resection materials of epilepsy patients with mesial temporal lobe sclerosis. J. Neurol. Sci..

[7] Love S., Perry A., Ironside J., Budka H. (2018). Greenfield′s Neuropathology-Two Volume Set.

[8] Brown R.C., Lockwood A.H., Sonawane B.R. (2005). Neurodegenerative diseases: An overview of environmental risk factors. Environ. Health Perspect..

[9] Churchill G.A., Airey D.C., Allayee H., Angel J.M., Attie A.D., Beatty J., Beavis W.D., Belknap J.K., Bennett B., Berrettini W. (2004). The Collaborative Cross, a community resource for the genetic analysis of complex traits. Nature Genet..

[10] Threadgill D.W., Hunter K.W., Williams R.W. (2002). Genetic dissection of complex and quantitative traits: From fantasy to reality via a community effort. Mamm. Genome.

[11] Noll K.E., Ferris M.T., Heise M.T. (2019). The Collaborative Cross: A systems genetics resource for studying host-pathogen interactions. Cell Host Microbe.

[12] Dal Canto M.C., Kim B.S., Miller S.D., Melvold R.W. (1996). Theiler′s murine encephalomyelitis virus (TMEV)-induced demyelination: A model for human multiple sclerosis. Methods.

[13] DePaula-Silva A.B., Hanak T.J., Libbey J.E., Fujinami R.S. (2017). Theiler′s murine encephalomyelitis virus infection of SJL/J and C57BL/6J mice: Models for multiple sclerosis and epilepsy. J. Neuroimmunol..

[14] Gerhauser I., Hansmann F., Ciurkiewicz M., Löscher W., Beineke A. (2019). Facets of theiler′s murine encephalomyelitis virus-induced diseases: An update. Int. J. Mol. Sci..

[15] Libbey J.E., Kirkman N.J., Smith M.C., Tanaka T., Wilcox K.S., White H.S., Fujinami R.S. (2008). Seizures following picornavirus infection. Epilepsia.

[16] Lawley K.S., Rech R.R., Elenwa F., Han G., Perez Gomez A.A., Amstalden K., Welsh C.J., Young C.R., Threadgill D.W., Brinkmeyer-Langford C.L. (2021). Host genetic diversity drives variable central nervous system lesion distribution in chronic phase of Theiler′s Murine Encephalomyelitis Virus (TMEV) infection. PLoS ONE.

[17] Westphal A., Cheng W., Yu J., Grassl G., Krautkrämer M., Holst O., Föger N., Lee K.-H. (2017). Lysosomal trafficking regulator Lyst links membrane trafficking to toll-like receptor–mediated inflammatory responses. J. Exp. Med..

[18] Feng T., Lacrampe A., Hu F. (2021). Physiological and pathological functions of TMEM106B: A gene associated with brain aging and multiple brain disorders. Acta Neuropathol..

[19] Ito Y., Hartley T., Baird S., Venkateswaran S., Simons C., Wolf N.I., Boycott K.M., Dyment D.A., Kernohan K.D. (2018). Lysosomal dysfunction in TMEM106B hypomyelinating leukodystrophy. Neurol. Genet..

[20] Klein R.S., Garber C., Funk K.E., Salimi H., Soung A., Kanmogne M., Manivasagam S., Agner S., Cain M. (2019). Neuroinflammation During RNA Viral Infections. Annu. Rev. Immunol..

[21] Van Sluijs L., Pijlman G.P., Kammenga J.E. (2017). Why do individuals differ in viral susceptibility? A story told by model organisms. Viruses.

[22] Eldridge R., Osorio D., Amstalden K., Edwards C., Young C.R., Cai J.J., Konganti K., Hillhouse A., Threadgill D.W., Welsh C.J. (2020). Antecedent presentation of neurological phenotypes in the Collaborative Cross reveals four classes with complex sex-dependencies. Sci. Rep..

[23] Brinkmeyer-Langford C.L., Rech R., Amstalden K., Kochan K.J., Hillhouse A.E., Young C., Welsh C.J., Threadgill D.W. (2017). Host genetic background influences diverse neurological responses to viral infection in mice. Sci. Rep..

[24] Buckwalter M.R., Nga P.T., Gouilh M.A., Fiette L., Bureau J.-F., Laird M.E., Buchrieser J., Ozden S., Cheval J., Eloit M. (2011). Identification of a novel neuropathogenic Theiler′s murine encephalomyelitis virus. J. Virol..

[25] Stewart K.A.A., Wilcox K.S., Fujinami R.S., White H.S. (2010). Theiler′s virus infection chronically alters seizure susceptibility. Epilepsia.

[26] Bröer S., Hage E., Käufer C., Gerhauser I., Anjum M., Li L., Baumgärtner W., Schulz T.F., Löscher W. (2017). Viral mouse models of multiple sclerosis and epilepsy: Marked differences in neuropathogenesis following infection with two naturally occurring variants of Theiler′s virus BeAn strain. Neurobiol. Dis..

[27] Kirkman N.J., Libbey J.E., Wilcox K.S., White H.S., Fujinami R.S. (2010). Innate but not adaptive immune responses contribute to behavioral seizures following viral infection. Epilepsia.

[28] Libbey J.E., Kennett N.J., Wilcox K.S., White H.S., Fujinami R.S. (2011). Lack of correlation of central nervous system inflammation and neuropathology with the development of seizures following acute virus infection. J. Virol..

[29] Loewen J.L., Barker-Haliski M.L., Dahle E.J., White H.S., Wilcox K.S. (2016). Neuronal Injury, Gliosis, and Glial Proliferation in Two Models of Temporal Lobe Epilepsy. J. Neuropathol. Exp. Neurol..

[30] Murray P., Pavelko K., Leibowitz J., Lin X., Rodriguez M. (1998). CD4+ and CD8+ T cells make discrete contributions to demyelination and neurologic disease in a viral model of multiple sclerosis. J. Virol..

[31] Lipton H.L. (1975). Theiler′s virus infection in mice: An unusual biphasic disease process leading to demyelination. Infect. Immun..

[32] Rock R.B., Gekker G., Hu S., Sheng W.S., Cheeran M., Lokensgard J.R., Peterson P.K. (2004). Role of microglia in central nervous system infections. Clin. Microbiol. Rev..

[33] Chhatbar C., Prinz M. (2021). The roles of microglia in viral encephalitis: From sensome to therapeutic targeting. Cell. Mol. Immunol..

[34] Waltl I., Käufer C., Gerhauser I., Chhatbar C., Ghita L., Kalinke U., Löscher W. (2018). Microglia have a protective role in viral encephalitis-induced seizure development and hippocampal damage. Brain Behav. Immun..

[35] Vargas G., Medeiros Geraldo L.H., Gedeao Salomao N., Viana Paes M., Regina Souza Lima F., Carvalho Alcantara Gomes F. (2020). Severe acute respiratory syndrome coronavirus 2 (SARS-CoV-2) and glial cells: Insights and perspectives. Brain Behav. Immun. Health.

[36] Luong N.H. (2020). Exosomes Secreted by Microglia Contribute to Virus Persistence and Demyelinating Disease. Ph.D. Thesis.

[37] Perez Gomez A.A., Karmakar M., Carroll R.J., Lawley K.S., Amstalden K., Young C.R., Threadgill D.W., Welsh C.J., Brinkmeyer-Langford C. (2021). Genetic and immunological contributors to virus-induced paralysis. Brain Behav. Immun. Health.

[38] Brinkmeyer-Langford C., Amstalden K., Konganti K., Hillhouse A., Lawley K., Perez-Gomez A., Young C.R., Welsh C.J., Threadgill D.W. (2021). Resilience in Long-Term Viral Infection: Genetic Determinants and Interactions. Int. J. Mol. Sci..

[39] Sanchez J.M.S., DePaula-Silva A.B., Doty D.J., Truong A., Libbey J.E., Fujinami R.S. (2019). Microglial cell depletion is fatal with low level picornavirus infection of the central nervous system. J. Neurovirol..

[40] Sheng L., Zhang W., Gu J., Shen K., Luo H., Yang Y. (2019). Novel mutations of STXBP2 and LYST associated with adult haemophagocytic lymphohistiocytosis with Epstein-Barr virus infection: A case report. BMC Med. Genet..

[41] Kuan M.I., Jaeger H.K., Balemba O.B., O′Dowd J.M., Duricka D., Hannemann H., Marx E., Teissier N., Gabrielli L., Bonasoni M.P. (2020). Human Cytomegalovirus Interactions with the Basement Membrane Protein Nidogen 1. J. Virol..

[42] Nicholson A.M., Rademakers R. (2016). What we know about TMEM106B in neurodegeneration. Acta Neuropathol..

[43] Baggen J., Persoons L., Vanstreels E., Jansen S., Van Looveren D., Boeckx B., Geudens V., De Man J., Jochmans D., Wauters J. (2021). Genome-wide CRISPR screening identifies TMEM106B as a proviral host factor for SARS-CoV-2. Nat. Genet..

[44] Ling S.C., Polymenidou M., Cleveland D.W. (2013). Converging mechanisms in ALS and FTD: Disrupted RNA and protein homeostasis. Neuron.

[45] Bignall K.E. (1974). Ontogeny of levels of neural organization: The righting reflex as a model. Exp. Neurol..

[46] Bose P., Fielding R., Ameis K.M., Vacca-Galloway L.L. (1998). A novel behavioral method to detect motoneuron disease in Wobbler mice aged three to seven days old. Brain Res..

[47] Johnson R.R., Storts R., Welsh T.H., Welsh C.J.R., Meagher M.W. (2004). Social stress alters the severity of acute Theiler′s virus infection. J. Neuroimmunol..

[48] Pappas S.S., Darr K., Holley S.M., Cepeda C., Mabrouk O.S., Wong J.-M.T., Lewitt T.M., Paudel R., Houlden H., Kennedy R.T. (2015). Forebrain deletion of the dystonia protein torsinA causes dystonic-like movements and loss of striatal cholinergic neurons. eLife.

[49] Kondori N.R., Paul P., Robbins J.P., Liu K., Hildyard J.C.W., Wells D.J., de Belleroche J.S. (2017). Characterisation of the pathogenic effects of the in vivo expression of an ALS-linked mutation in D-amino acid oxidase: Phenotype and loss of spinal cord motor neurons. PLoS ONE.

[50] Burrer R., Buchmeier M.J., Wolfe T., Ting J.P.C., Feuer R., Iglesias A., Von Herrath M.G. (2007). Exacerbated Pathology of Viral Encephalitis in Mice with Central Nervous System-Specific Autoantibodies. Am. J. Pathol..

[51] Campbell T., Meagher M.W., Sieve A., Scott B., Storts R., Welsh T.H., Welsh C.J.R. (2001). The Effects of Restraint Stress on the Neuropathogenesis of Theiler′s Virus Infection: I. Acute Disease. Brain Behav. Immun..

[52] Sieve A.N., Steelman A.J., Young C.R., Storts R., Welsh T.H., Welsh C.J.R., Meagher M.W. (2004). Chronic restraint stress during early Theiler′s virus infection exacerbates the subsequent demyelinating disease in SJL mice. J. Neuroimmunol..

[53] Köhler C. (2007). Allograft inflammatory factor-1/Ionized calcium-binding adapter molecule 1 is specifically expressed by most subpopulations of macrophages and spermatids in testis. Cell Tissue Res..

[54] Bertram C.A., Klopfleisch R. (2017). The pathologist 2.0: An update on digital pathology in veterinary medicine. Vet. Pathol..

[55] Riber-Hansen R., Vainer B., Steiniche T. (2012). Digital image analysis: A review of reproducibility, stability and basic requirements for optimal results. Apmis.

[56] Konganti K., Ehrlich A., Rusyn I., Threadgill D.W. (2018). gQTL: A Web Application for QTL Analysis Using the Collaborative Cross Mouse Genetic Reference Population. G3 Genes Genomes Genet..

