# Supplementary material for: Viral Clearance and Neuroinflammation in Acute TMEV Infection Vary by Host Genetic Background
Source: Int J Mol Sci. 2022 Sep 9;23(18):10482. doi: 10.3390/ijms231810482 (PMC9501595; doi:10.3390/ijms231810482)
Supplement: Supplementary file 1 [file ijms-23-10482-s001.zip › ijms-1882487-supplementary.pdf]

Supplemental Figures and Tables

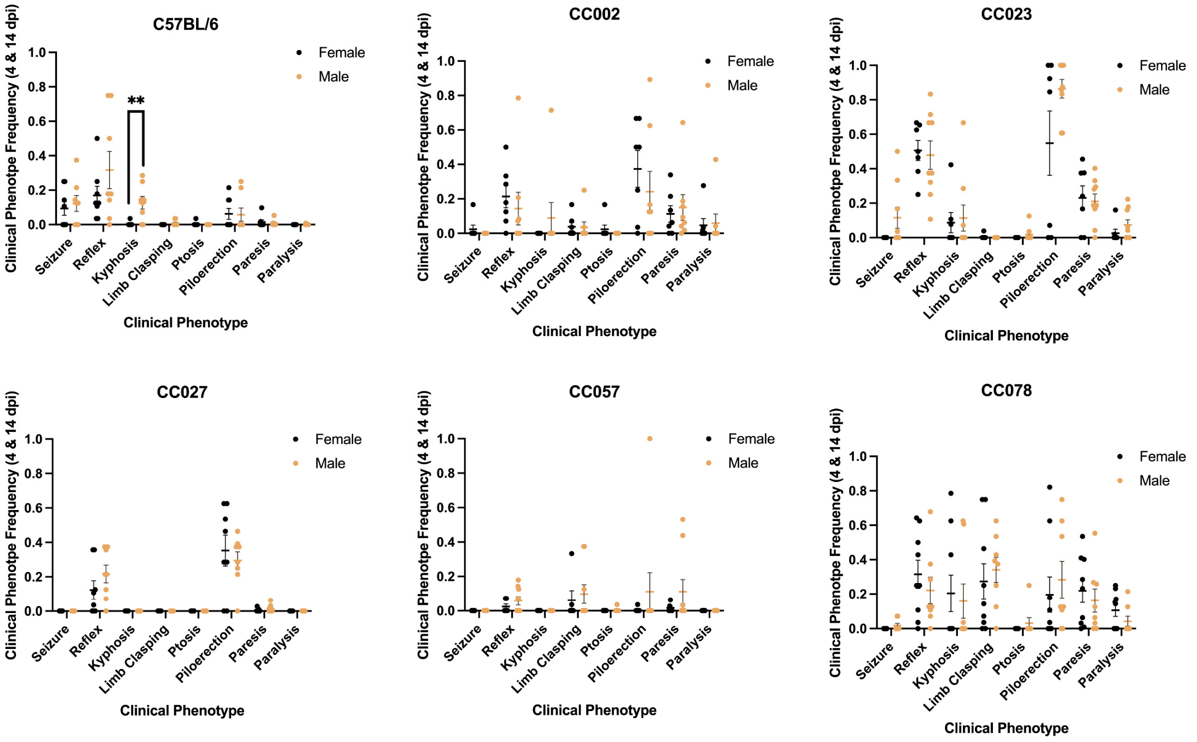

Supplemental Figure S1. Female and male mice did not exhibit significant differences in TMEV-induced clinical signs during the acute phase of viral infection. Data shown here are the mean ± SEM of the average clinical phenotype frequency for each strain with 4 and 14 dpi timepoints being pooled. P values were determined using the Wilcoxon rank sum tests *, P<0.05; **, p<0.01; ***, P<0.001.

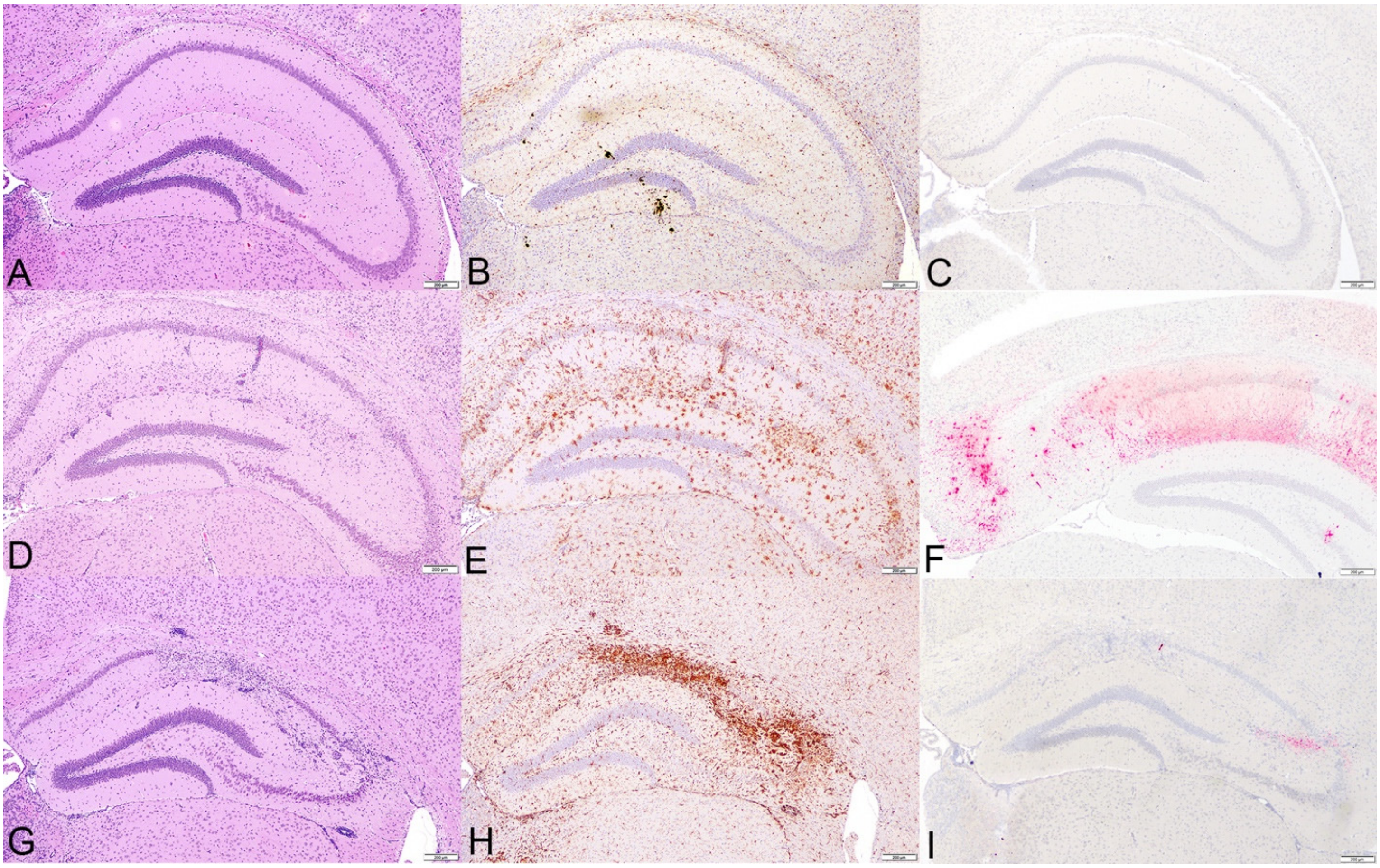

Supplemental Figure S2. Cross sections of the hippocampal formation at level B of C57BL/6J mice infected with Theiler's Murine Encephalomyelitis Virus (TMEV). A, B, C. Sham mouse. A. Normal hippocampal formation. H&E stain. B. Iba-1 immunolabeling. This image has black clumps from the automated stainer over the tissue, which were interpreted as artifact. C. RNA *in situ* hybridization for TMEV with lack of mRNA nuclear expression. D, E, F. Infected mouse euthanized at 4 dpi. D. Neuroparenchymal gliosis and perivascular cuffing of the stratum radiatum, stratum lacunosum-moleculare and stratum oriens with mild neuronal necrosis of CA2 pyramidal layer. H&E stain. E. Across the hippocampal formation the glial reaction and perivascular cuffing in D corresponded to Iba-1 immunolabeling. F. TMEV mRNA expression

was located in the hippocampal formation and in the retrosplenial area of the cortex. G, H, I. Infected mouse euthanized at 14 dpi. G. Locally extensive neuronal necrosis and loss of CA1, focal neuronal necrosis and loss of CA2 pyramidal layers with marked gliosis and perivascular cuffing of stratum radiatum, stratum lacunosum-moleculare and stratum oriens. H. Across the hippocampal formation the glial reaction and perivascular cuffing in G corresponded to strong Iba-1 immunolabeling. I. TMEV mRNA expression was mostly cleared by 14 dpi and was located at field CA2. Bar=200 µm.

Table S1. Sample sizes of sham and infected mice used for each timepoint.

	**Infected Status**	**Sham**		**Infected**	
	**Sex**	**Male**	**Female**	**Male**	**Female**
4 DPI	C57BL/6	3	3	4	4
	CC002	3	5	4	4
	CC023	6	3	6	4
	CC027	6	4	4	4
	CC057	3	4	4	2
	CC078	3	3	4	3
14 DPI	C57BL/6	4	4	4	4
	CC002	3	3	5	3
	CC023	3	3	3	3
	CC027	3	3	4	4
	CC057	3	3	5	4

	CC078	3	3	4	6

Table S2: Strain C57BL/6 descriptive statistics for 4 and 14 days post infection for infected mice

	DPI 4				DPI 14			
	N	M	Median	IQR	N	M	Median	IQR
Clinical Phenotype								
Seizure	8	0.1094	0.0625	0.1875	8	0.1071	0.1250	0.1786
Reflex	8	0.3750	0.3750	0.500	8	0.1160	0.1250	0.1429
Kyphosis	8	0.0469	0.0000	0.0625	8	0.0848	0.0536	0.1429
Limb Clasping	8	0.0000	0.0000	0.0000	8	0.0089	0.0000	0.0179
Ptosis	8	0.0000	0.0000	0.0000	8	0.0045	0.0000	0.0000
Piloerection	8	0.0000	0.0000	0.0000	8	0.1205	0.1429	0.2143
Paresis	8	0.0000	0.0000	0.0000	8	0.0246	0.0089	0.0312
Paralysis	8	0.0000	0.0000	0.0000	8	0.0011	0.0000	0.0000

Table S3: Strain CC002 descriptive statistics for 4 and 14 days post infection for infected mice

	DPI 4				DPI 14			
	N	M	Median	IQR	N	M	Median	IQR
Clinical Phenotype								
Seizure	8	0.0208	0.0000	0.0000	8	0.0000	0.0000	0.0000
Reflex	8	0.1198	0.0000	0.2292	8	0.2195	0.1310	0.1756
Kyphosis	8	0.0000	0.0000	0.0000	8	0.0893	0.0000	0.0000
Limb Clasping	8	0.0521	0.0000	0.0833	8	0.0179	0.0000	0.0357
Ptosis	8	0.0208	0.0000	0.0000	8	0.0000	0.0000	0.0000
Piloerection	8	0.4010	0.5000	0.5208	8	0.2128	0.1012	0.3036
Paresis	8	0.0521	0.0521	0.0781	8	0.2619	0.2009	0.3408
Paralysis	8	0.000	0.0000	0.0000	8	0.1179	0.0387	0.2009

Table S4: Strain CC023 descriptive statistics for 4 and 14 days post infection for infected mice

	DPI 4				DPI 14			
	N	M	Median	IQR	N	M	Median	IQR
Clinical Phenotype								
Seizure	8	0.0208	0.0000	0.0000	8	0.0000	0.0000	0.0000
Reflex	8	0.1198	0.0000	0.2292	8	0.2195	0.1310	0.1756
Kyphosis	8	0.0000	0.0000	0.0000	8	0.0893	0.0000	0.0000
Limb Clasping	8	0.0521	0.0000	0.0833	8	0.0179	0.0000	0.0357
Ptosis	8	0.0208	0.0000	0.0000	8	0.0000	0.0000	0.0000
Piloerection	8	0.4010	0.5000	0.5208	8	0.2128	0.1012	0.3036

Paresis	8	0.0521	0.0521	0.0781	8	0.2619	0.2009	0.3408
Paralysis	8	0.0000	0.0000	0.0000	8	0.1179	0.0387	0.2009

Table S5: Strain CC027 descriptive statistics for 4 and 14 days post infection for infected mice

	DPI 4				DPI 14			
	N	M	Median	IQR	N	M	Median	IQR
Clinical Phenotype								
Seizure	8	0.0000	0.0000	0.0000	8	0.0000	0.0000	0.0000
Reflex	8	0.1250	0.0625	0.2500	8	0.2143	0.2143	0.2678
Kyphosis	8	0.0000	0.0000	0.0000	8	0.0000	0.0000	0.0000
Limb Clasping	8	0.0000	0.0000	0.0000	8	0.0000	0.0000	0.0000
Ptosis	8	0.0000	0.0000	0.0000	8	0.0000	0.0000	0.0000
Piloerection	8	0.2813	0.3125	0.5000	8	0.3661	0.3393	0.1786
Paresis	8	0.0117	0.0000	0.0156	8	0.0078	0.0045	0.0134
Paralysis	8	0.0000	0.0000	0.0000	8	0.0000	0.0000	0.0000

Table S6: Strain CC057 descriptive statistics for 4 and 14 days post infection for infected mice

	DPI 4				DPI 14			
	N	M	Median	IQR	N	M	Median	IQR
Clinical Phenotype								
Seizure	6	0.0000	0.0000	0.0000	9	0.0000	0.0000	0.0000
Reflex	6	0.2080	0.0000	0.0000	9	0.0595	0.0714	0.0714
Kyphosis	6	0.0000	0.0000	0.0000	9	0.0000	0.0000	0.0000
Limb Clasping	6	0.2014	0.2292	0.3750	9	0.0040	0.0000	0.0000
Ptosis	6	0.0000	0.0000	0.0000	9	0.0040	0.0000	0.0000
Piloerection	6	0.1667	0.0000	0.0000	9	0.0040	0.0000	0.0000
Paresis	6	0.1719	0.0313	0.4375	9	0.0040	0.0000	0.0000
Paralysis	6	0.0000	0.0000	0.0000	9	0.0000	0.0000	0.0000

Table S7: Strain CC078 descriptive statistics for 4 and 14 days post infection for infected

	DPI 4				DPI 14			
	N	M	Median	IQR	N	M	Median	IQR
Clinical Phenotype								
Seizure	7	0.0000	0.0000	0.0000	10	0.0143	0.0000	0.0000
Reflex	7	0.2143	0.1250	0.3750	10	0.3107	0.2679	0.3929
Kyphosis	7	0.1786	0.0000	0.6250	10	0.1857	0.0000	0.0000
Limb Clasping	7	0.3036	0.2500	0.6250	10	0.3071	0.3214	0.3929
Ptosis	7	0.0000	0.0000	0.0000	10	0.0250	0.0000	0.0000
Piloerection	7	0.3214	0.1250	0.6250	10	0.1786	0.0714	0.1786

Paresis	7	0.0446	0.0313	0.0625	10	0.2955	0.7568	0.1786
Paralysis	7	0.0000	0.0000	0.0000	10	0.1286	0.1473	0.2143

Table S8: Strain C57BL/6 descriptive statistics for male and female for infected mice

	Male				Female			
	N	M	Median	IQR	N	M	Median	IQR
Clinical Phenotype								
Seizure	8	0.1228	0.1250	0.1786	8	0.0938	0.0536	0.1964
Reflex	8	0.3170	0.1786	0.5357	8	0.1696	0.1250	0.1429
Kyphosis	8	0.1272	0.1339	0.1617	8	0.0045	0.0000	0.0000
Limb Clasping	8	0.0089	0.0000	0.0179	8	0.0000	0.0000	0.0000
Ptosis	8	0.0000	0.0000	0.0000	8	0.0045	0.0000	0.0000
Piloerection	8	0.0580	0.0000	0.1071	8	0.0625	0.0000	0.1429
Paresis	8	0.0089	0.0000	0.0089	8	0.0156	0.0000	0.0134
Paralysis	8	0.0011	0.0000	0.0000	8	0.0000	0.0000	0.0000

Table S9: Strain CC002 descriptive statistics for male and female for infected mice

	Male				Female			
	N	M	Median	IQR	N	M	Median	IQR
Clinical Phenotype								
Seizure	9	0.0000	0.0000	0.0000	7	0.0238	0.0000	0.0000
Reflex	9	0.1356	0.0714	0.0833	7	0.2134	0.1786	0.2470
Kyphosis	9	0.0794	0.0000	0.0000	7	0.0000	0.0000	0.0000
Limb Clasping	9	0.0317	0.0000	0.0000	7	0.0317	0.0000	0.0000
Ptosis	9	0.0000	0.0000	0.0000	7	0.0000	0.0000	0.0000
Piloerection	9	0.2546	0.1250	0.3751	7	0.3741	0.5000	0.6310
Paresis	9	0.1911	0.0938	0.1458	7	0.1131	0.0625	0.2143
Paralysis	9	0.0681	0.0000	0.0417	7	0.0472	0.0000	0.0357

Table S10: Strain CC023 descriptive statistics for male and for infected mice

	Male				Female			
	N	M	Median	IQR	N	M	Median	IQR
Clinical Phenotype								
Seizure	9	0.1151	0.0000	0.1667	7	0.0000	0.0000	0.0000
Reflex	9	0.4788	0.3750	0.2692	7	0.5063	0.5000	0.3453
Kyphosis	9	0.1138	0.0000	0.0714	7	0.0867	0.0000	0.1071
Limb Clasping	9	0.0000	0.0000	0.0000	7	0.0000	0.0000	0.0000
Ptosis	9	0.0179	0.0000	0.0000	7	0.0000	0.0000	0.0000

Piloerection	9	0.8644	0.8750	0.1667	7	0.5487	0.8461	1.0000
Paresis	9	0.2102	0.1875	0.1279	7	0.2306	0.2404	0.3765
Paralysis	9	0.0741	0.0417	0.1607	7	0.0257	0.0000	0.1607

Table S11: Strain CC027 descriptive statistics for male and female for infected mice

	Male				Female			
	N	M	Median	IQR	N	M	Median	IQR
Clinical Phenotype								
Seizure	8	0.0000	0.0000	0.0000	8	0.0000	0.0000	0.0000
Reflex	8	0.2165	0.2143	0.2769	8	0.1228	0.0714	0.2411
Kyphosis	8	0.0000	0.0000	0.0000	8	0.0000	0.0000	0.0000
Limb Clasping	8	0.0000	0.0000	0.0000	8	0.0000	0.0000	0.0000
Ptosis	8	0.0000	0.0000	0.0000	8	0.0000	0.0000	0.0000
Piloerection	8	0.2946	0.3304	0.1518	8	0.3527	0.3750	0.4375
Paresis	8	0.0139	0.0000	0.0246	8	0.0056	0.0000	0.0089
Paralysis	8	0.0000	0.0000	0.0000	8	0.0000	0.0000	0.0000

Table S12: Strain CC057 descriptive statistics for male and female for infected mice

	Male				Female			
	N	M	Median	IQR	N	M	Median	IQR
Clinical Phenotype								
Seizure	9	0.0000	0.0000	0.0000	6	0.0000	0.0000	0.0000
Reflex	9	0.0595	0.0714	0.0714	6	0.0238	0.0000	0.0714
Kyphosis	9	0.0000	0.0000	0.0000	6	0.0000	0.0000	0.0000
Limb Clasping	9	0.0972	0.0000	0.0125	6	0.0615	0.0000	0.0000
Ptosis	9	0.0040	0.0000	0.0000	6	0.0000	0.0000	0.0000
Piloerection	9	0.1111	0.0000	0.0000	6	0.0059	0.0000	0.0000
Paresis	9	0.1111	0.0000	0.0313	6	0.0164	0.0045	0.0268
Paralysis	9	0.0000	0.0000	0.0000	6	0.0000	0.0000	0.0000

Table S13: Strain CC078 descriptive statistics for male and female for infected mice

	Male				Female			
	N	M	Median	IQR	N	M	Median	IQR
Clinical Phenotype								
Seizure	8	0.0179	0.0000	0.0357	9	0.0000	0.0000	0.0000
Reflex	8	0.2210	0.1250	0.2410	9	0.3155	0.2500	0.3929
Kyphosis	8	0.1585	0.0000	0.3214	9	0.2044	0.0000	0.4286
Limb Clasping	8	0.3415	0.3839	0.2946	9	0.2738	0.1429	0.4286
Ptosis	8	0.0313	0.0000	0.0000	9	0.0000	0.0000	0.0000

Piloerection	8	0.2835	0.1250	0.5268	9	0.1964	0.0357	0.1786
Paresis	8	0.1624	0.0938	0.2478	9	0.2188	0.2321	0.3705
Paralysis	8	0.0424	0.0000	0.0625	9	0.1052	0.1429	0.1696
